# Supplementary figures and images for: A defining member of the new cysteine-cradle family is an aECM protein signalling skin damage in C. elegans
Source: PLoS Genet. 2025 Mar 20;21(3):e1011593. doi: 10.1371/journal.pgen.1011593 (PMC11925461; doi:10.1371/journal.pgen.1011593)

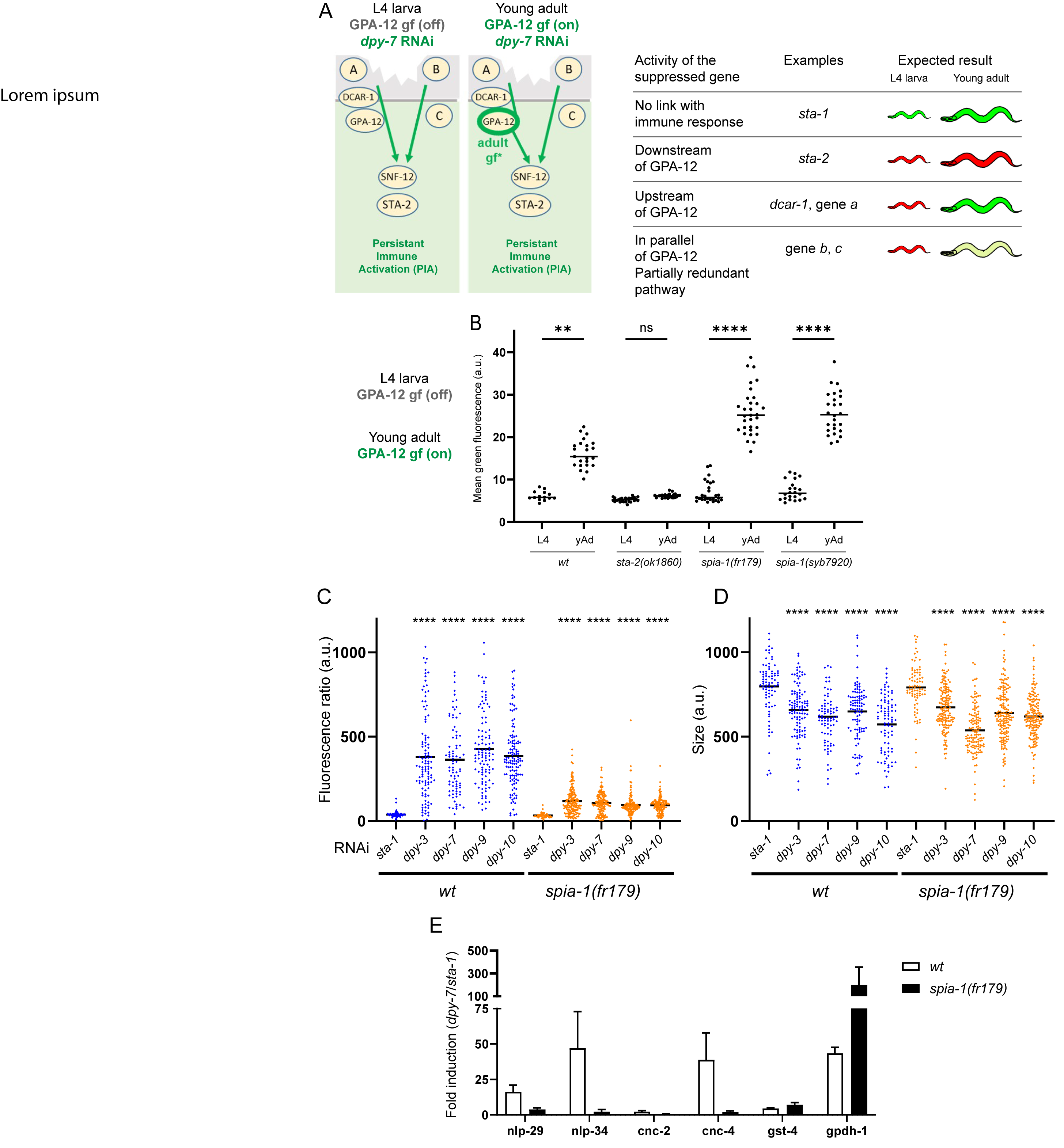

Supplement: S1 Fig — (A) In the suppressor screen, we triggered a PIA in the strain IG1389 by inactivating dpy-7 by RNAi (left). In this strain, the state of the immune response is monitored (frIs7 construct; green fluorescence off=inactive, green fluorescence on=active) and GPA-12 is constitutively active in the adult (frIs30). Different scenarios are expected depending on the gene affected after EMS-induced mutagenesis (right). (B) Quantification of relative green fluorescence in worms carrying frIs7 and frIs30 constructs, but without dpy-7 RNAi inactivation, in L4 and young adults (yAd); n>14. Only the inactivation of a gene acting downstream of GPA-12 (e.g., sta-2) leads to the suppression of the green fluorescence in adults. (C-D) Quantification with the Biosort of the ratio between nlp-29p::GFP intensity and size (C) and of the size of the worms (D) in wt or spia-1(fr179) adults following RNAi inactivation of the 4 furrow collagen genes and the sta-1 control; n>70, one of 3 independent experiments. spia-1(fr179) does not suppress the short size induced via inactivation of the 4 furrow collagen genes. Statistical comparisons were made by comparing to the corresponding sta-1 control. **p < 0.01; ****p < 0.0001. (E) mRNA levels of nlp-29, nlp-34, cnc-2, cnc-4, gst-4 and gpdh-1 were quantified by qPCR in wild-type and spia-1(fr179) worms upon RNAi inactivation of sta-1 or dpy-7, in three independent experiments. The mean fold-changes between the dpy-7 and sta-1 levels are represented. In spia-1(fr179), the transcription of AMPs genes including nlp-29, nlp-34 and cnc-4 were reduced, contrary to the transcription of gst-4 and gpdh-1, the latter being increased. (TIF) [file pgen.1011593.s001.tif]

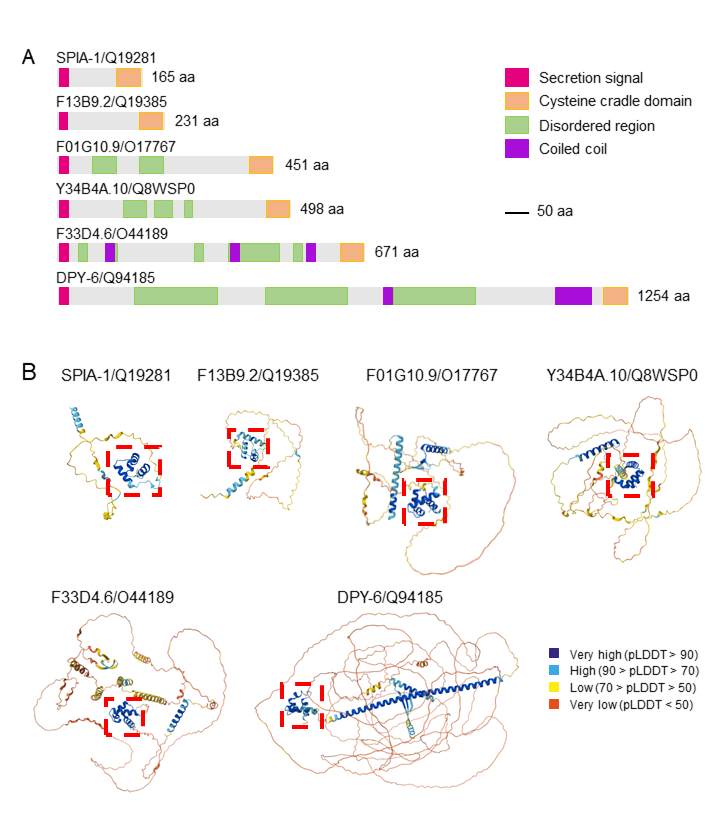

Supplement: S2 Fig — (A) Domain organisation of the 6 CCD-aECM proteins in C. elegans, as annotated in InterPro [28,64] and (B) structural models predicted with AlphaFold [27,65], rendered with the Predicted Local Distance Difference Test score (pLDDT), which indicates how well a predicted protein structure matches protein data bank structure information and multiple sequence alignment data. (TIF) [file pgen.1011593.s002.tif]

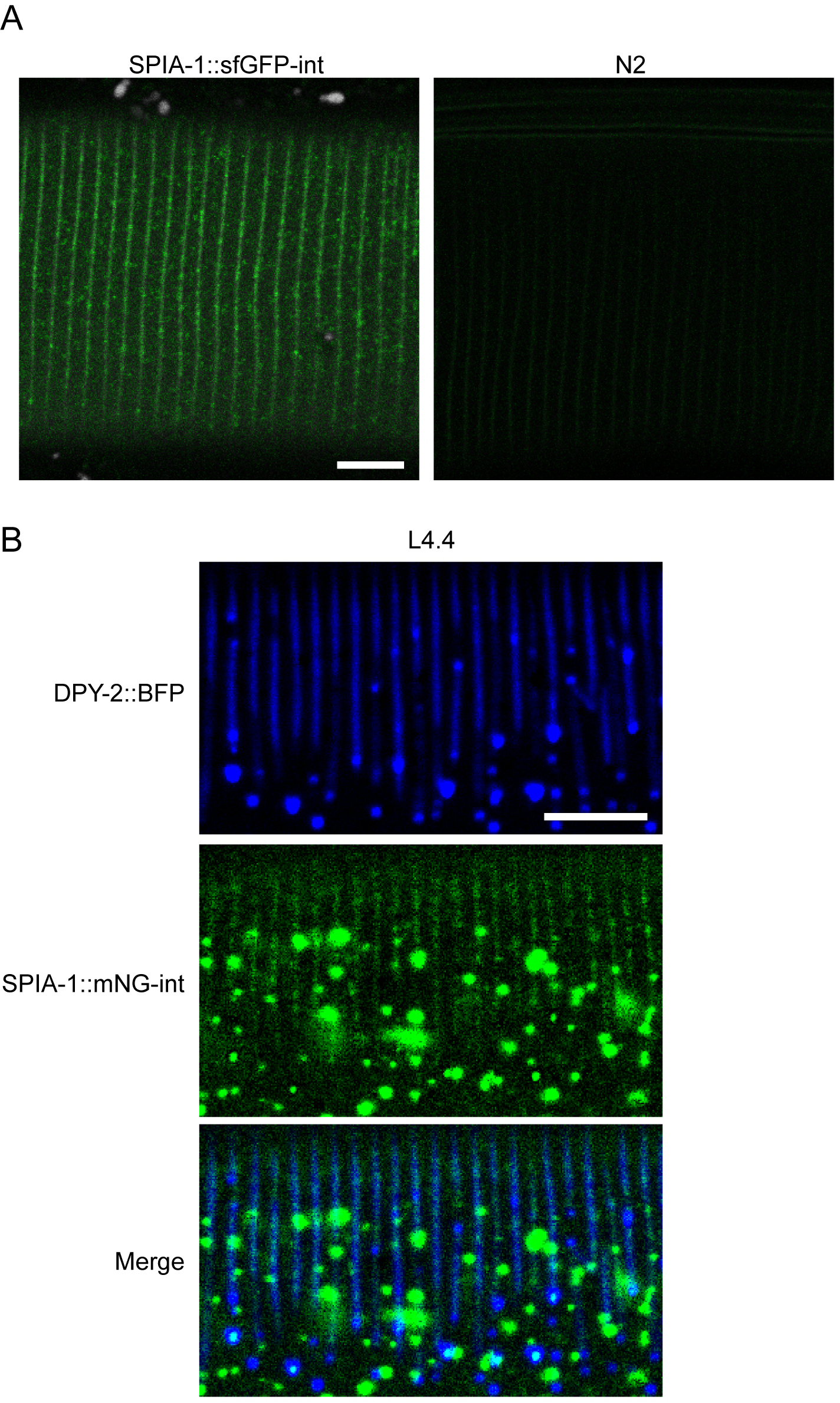

Supplement: S3 Fig — (A) The confocal image of the SPIA-1::sfGFP reporter (GFP-int) in the adult shown in Fig 4D is presented aside from a representative adult wild-type imaged using same illumination conditions; n>5, scale bar, 5 µm. (B) Zoom on the furrows in the L4.4 shown in Fig 4F. Both single channels and the merge are shown, as depicted. NUC-1::mCherry is not shown for clarity; scale bar, 5 µm. (TIF) [file pgen.1011593.s003.tif]

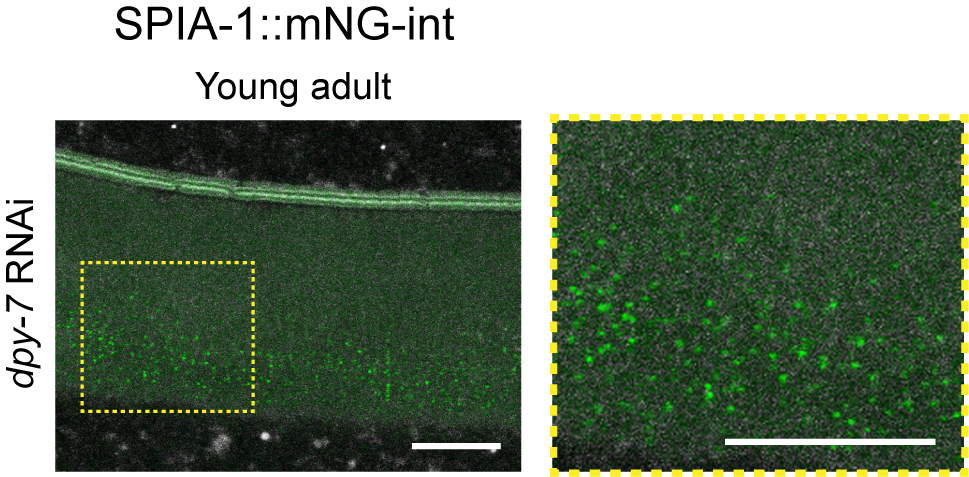

Supplement: S4 Fig — Representative images of SPIA-1::mNG-int young adults following dpy-7 RNAi inactivation. To compare with Fig 5E. A ~2.5 times magnification of the area contained in the dashed rectangle is provided on the far right; n>5, scale bar, 10 µm. (TIF) [file pgen.1011593.s004.tif]

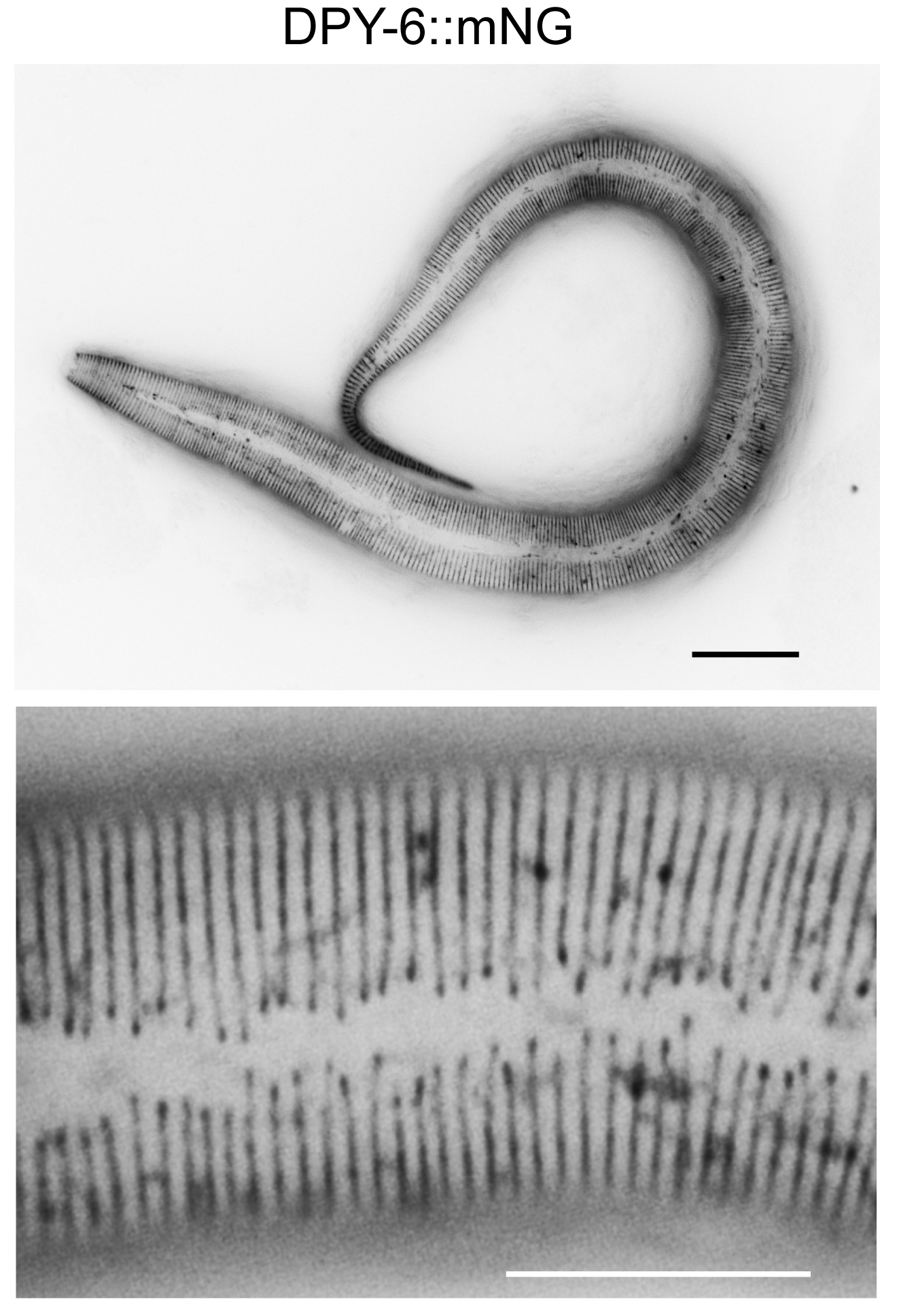

Supplement: S5 Fig — Representative fluorescent images of the furrow localisation of DPY-6::mNG-int, in a L1 (top) or L2 larva (bottom); n>5, scale bar, 20 µm (top), 10 µm (bottom). (TIF) [file pgen.1011593.s005.tif]
